# Author Correction: Searching thousands of genomes to classify somatic and novel structural variants using STIX

**DOI:** 10.1038/s41592-022-01538-8

**Published:** 2022-05-26

**Authors:** Murad Chowdhury, Brent S. Pedersen, Fritz J. Sedlazeck, Aaron R. Quinlan, Ryan M. Layer

**Affiliations:** 1 BioFrontiers Institute, University of Colorado, Boulder, CO USA; 2 University Medical Center, Utrecht University, Utrecht, the Netherlands; 3 Human Genome Sequencing Center, Baylor College of Medicine, Houston, TX USA; 4 Department of Human Genetics, University of Utah, Salt Lake City, UT USA; 5 Department of Biomedical Informatics, University of Utah, Salt Lake City, UT USA; 6 Utah Center for Genetic Discovery, University of Utah, Salt Lake City, UT USA; 7 Department of Computer Science, University of Colorado, Boulder, CO USA

**Keywords:** Software, Genome informatics, Software, Genomics, Mutation

Correction to: *Nature Methods* 10.1038/s41592-022-01423-4, published online 8 April 2022.

In the version of this article initially published, the Acknowledgements section for Ryan M. Layer did not include the funding information “NIH/NCI grant no. UO1 CA231978.” The grant has been included in the HTML and PDF versions of the article.

