# Supplementary material for: Searching thousands of genomes to classify somatic and novel structural variants using STIX
Source: Nat Methods. 2022 Apr 8;19(4):445–8. doi: 10.1038/s41592-022-01423-4 (PMC9007735; doi:10.1038/s41592-022-01423-4)
Supplement: Supplementary file 1 — Supplementary Notes 1–4, Figs. 1–4 and Tables 1–3 and 5. [file 41592_2022_1423_MOESM1_ESM.pdf]

nature portfolio

https://doi.org/10.1038/s41592-022-01423-4

Supplementary information

Searching thousands of genomes to classify somatic and novel structural variants using STIX

In the format provided by the authors and unedited

Supplementary Notes

1. Challenges with calling and genotyping SVs

Part of the challenge when moving from SNVs to SVs is the substantial increase in the uncertainty of the underlying data. For example, the allele balance for heterozygous SNVs and SVs from the Genome In a Bottle Consortium[1,2] sample shows a shift from the expected peak at 0.5 allele balance in SNVs (**Fig. 1A**) to 0.3 in SVs (**Fig. 1B**). The reason for this shift is that SV detection and genotyping from short-read data is complicated by evidence that does not provide direct information about the location of the variant (e.g., read depth and discordant pair-reads). These two issues result in fundamentally different detection and genotyping strategies for SVs. Instead of explicitly testing for the existence of every possible SV (which is intractable), read alignment evidence is clustered, and a consensus breakpoint (which is often not at single-base resolution) and genotype is inferred. The two major issues with this type of clustering are instances where spurious alignments overlap by chance, causing false positives, and where fluctuations in coverage create false negatives or incorrect genotypes. Both of these cases produce SVs with a wide range of per-sample evidence depths and summarizing each sample into just three states (homozygous reference, heterozygous, and homozygous alternate) hides information that can be important when determining if a newly observed variant is common, rare, or noise. Genotype quality scores capture some of this uncertainty, but in practice, these scores are only used to exclude problematic samples from an analysis. This highlights the need for new metrics that can represent the full extent of structural variant evidence in a population.

2. COSMIC/PCAWG SVs present in STIX 1KG/SGDP database

Given its scalability, we can use STIX to improve somatic SV calls by scanning thousands of genomes for corroborating evidence. Among the 46,185 deletions in the Catalogue of Somatic Mutations in Cancer[3] (COSMIC), 12,270 (26.5%) appeared in the 1KG STIX database (**Fig. 2A**), 12,902 (27.9%) were in the SGDP STIX database (**Fig. 2B**), and 13,295 (28.8%) were in the combined cohort database (see **Supplementary Table 2**). Despite having matched normal tissues for every sample, 1,732 (2.1%) of the 84,083 somatic deletions found by PCAWG were in 1KG (**Fig. 2D**), 2,833 (3.4%) were in SGDP (**Fig. 2E**), and 3,237 (3.8%) appeared in either population (see **Supplementary Table 3**). The SVs found by STIX are likely either germline or recurrent mutations and are unlikely to be driving tumor evolution. These results highlight the importance of using STIX for future studies to incorporate larger reference populations to prioritize SVs.

Scanning a large population for recurring SVs can improve somatic calling, but relying on an SV call set of the population is insufficient. While STIX found that the 12,270 COSMIC SVs had some evidence in the 1KG cohort, the published 1KG SV call set[4] only recovered 454 variants (**Fig. 2C**). Similarly, only 193 PCAWG variants were in the 1KG catalog versus the 1,668 found by STIX (**Fig. 2F**), and many of the missing SVs were at high frequency (x=0 for **Figs. 2C** and **2F**). SV calls from larger cohorts are also less sensitive. For example, gnomAD SV[5], which included 14,918 genomes, only found 893 COSMIC SVs and 433 PCAWG SVs.

3. De novo variation STIX analysis

In addition to somatic SVs, we used STIX to study *de novo* variation in a large family study [6]. Since *de novo* SVs are new events, they should be rare in the population if mutations arise largely at random. Our analysis found strong evidence (at least three supporting reads) for 57 of 698 *de novo* SVs in either 1KG or SGDP (8.7% deletions, 5.6% duplications, 30% inversions) (see **Supplementary Table 5**). Most (47) *de novo* SVs were observed in a single 1KG sample, and one was in six. Given the massive number of possible SV combinations, the low *de novo* SV rate (0.16 events per genome[6]), and the likelihood that these SV are true *de novo* variants, finding any evidence in these populations highlights the plausibility of recurring alleles, which has been shown in other species[7], and in some complex diseases[8]. Only five of the reported *de novo* deletions appear in the 1KG catalog. STIX again shows its utility and importance in uncovering novel insights into SV dynamics by enabling an accessible and comprehensive assessment from population data for variants often not reported in SV catalogs.

4. STIX query resolution

When a paired-end read spanning an SV breakpoint is aligned to a reference genome, it will often have a notably different configuration from the vast majority of the other paired-end read alignments. For example, the ends of a pair spanning a deletion will align to loci that are further apart than expected. While these “discordant pairs" are a primary signal for short-read SV callers, they only convey indirect evidence of an SV since the breakpoint is not sequenced by either end. The result is ambiguity in the exact breakpoint location. STIX also uses discordant pairs when assessing the number of samples that contain evidence supporting an SV, and the uncertainty inherent to the evidence affects the resolution of the results. For example, queries against the 1KG cohort have a resolution between 200bp and 400bp (which is close to the insert size mean) (**Supplementary Figure 4**). The resolution of split-read evidence is better since the breakpoint is fully sequenced and can be more accurately localized.

Supplementary Figures

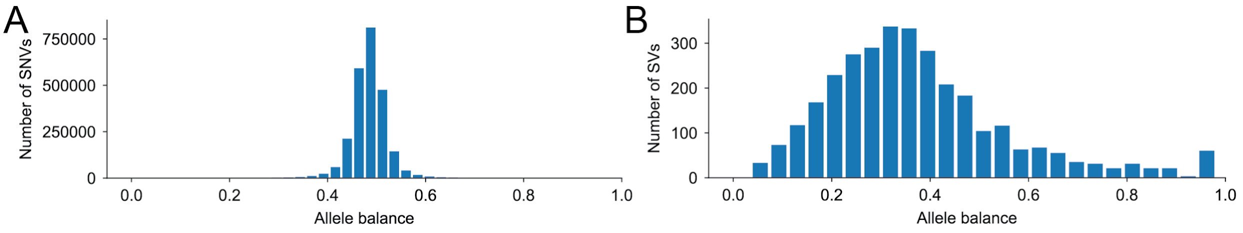

Supplementary Figure 1. Allele balance (number of reads matching the reference/total reads) for (**A**) SNVs and (**B**) SVs for the HG002 individual from the Genome in a Bottle Consortium.

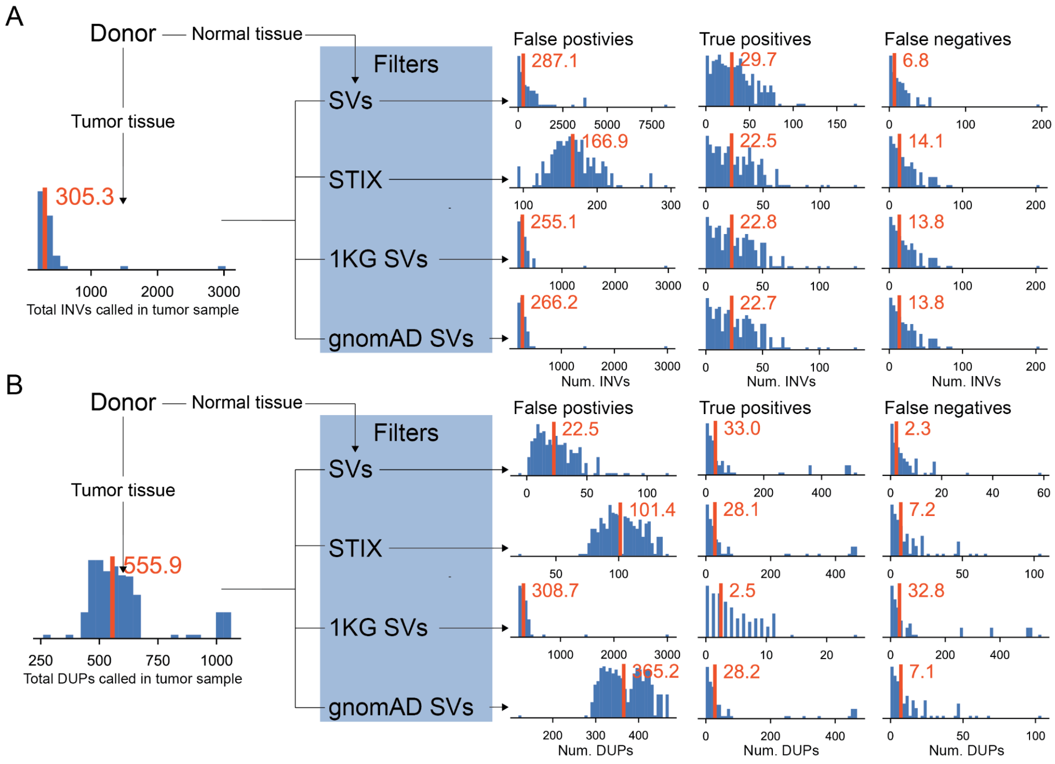

Supplementary Figure 2. A comparison of germline (A) inversion and (B) duplication filtering strategies for 183 prostate tumor samples that remove tumor deletions found in: matched-normal tissue (SV), the STIX index of 1KG, the 1KG SV calls, and the gnomAD SV calls.

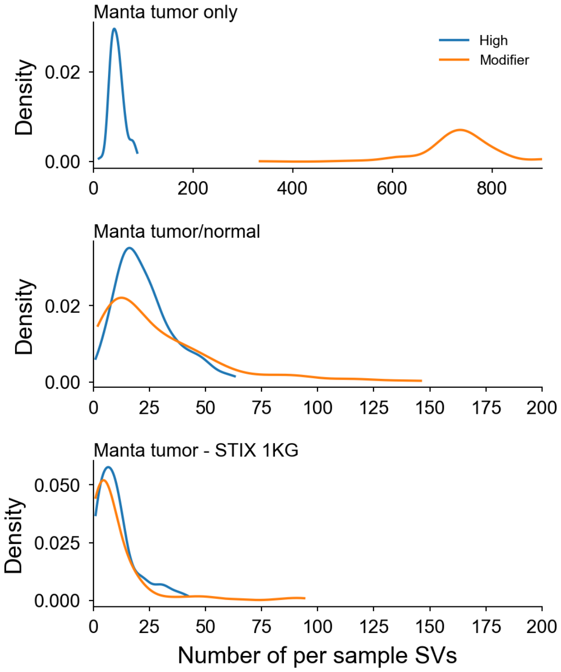

Supplementary Figure 3. The density of VEP annotation types in tumor-only SV calls, somatic calls that incorporated normal tissue, and tumor SVs filtered using the 1KG STIX index. VEP annotated SVs that are predicted to affect gene function as HIGH, and annotated those that don't as MODIFIER. The average per-sample number of SVs annotated as MODIFIER and HIGH in the tumor, tumor/normal, and STIX-filtered calls were 735.0 and 47.5, 28.6 and 22.8, and 10.0 and 10.5, respectively.

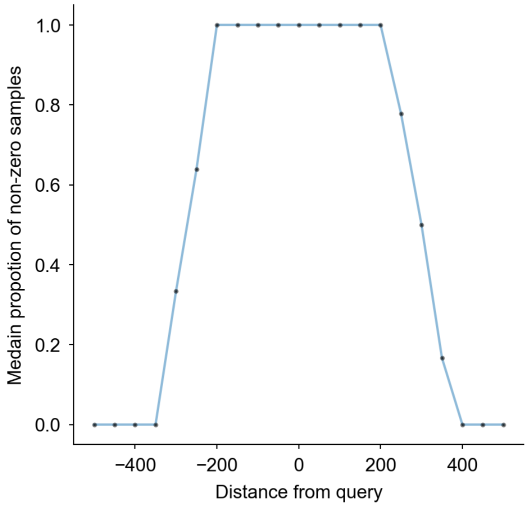

Supplementary Figure 4. STIX query resolution depends on the insert size distribution of the cohort under consideration. By shifting the query coordinates of 28,593 deletions called by 1KG up and downstream in 50bp increments and recalculating the number of samples found to still have evidence for the SV, we find the 1KG STIX queries have a resolution about about 400bp.

Supplementary Tables

	Deletions	Duplications	Inversions
number tested	32,021	365	786
accuracy	0.989	0.995	0.988
precision	0.955	0.135	0.962
sensitivity	0.645	0.514	0.713
specificity	0.999	0.996	0.999
F1	0.770	0.213	0.819

Supplementary Table 1. STIX performance across SV types considering the 1KG SV calls. In general, STIX performed well across all SV types and did exceptionally well for accuracy, precision, and specificity. The one exception was that STIX had a high number of false-positive duplication calls, leading to low precision and sensitivity. Upon inspection, just seven loci accounted for 95% of the false-positive calls. For these duplications, STIX estimated a much higher population frequency than what was listed in the 1KG catalog.

	Deletions	Duplications	Inversions
COSMIC SV catalog	46185	8904	18830
STIX SGDP	12902	58	828
STIX 1KG	12270	23	802
STIX SGDP + 1KG	13295	78	1006
1KG SV catalog	454	5	11
gnomAD SV catalog	893	26	50

Supplementary Table 2. The frequency of purportedly somatic SVs from the COSMIC database considering different SV collections. STIX consistently found evidence for many more COSMIC SVs than other sources even when considering the same underlying samples (i.e., STIX 1KG versus the 1KG SV catalog).

	Deletions	Duplications	H2H Inversions	T2T Inversions
PCAWG catalog	84083	72764	38602	37613
STIX SGDP	2833	790	3091	2893
STIX 1KG	1732	221	2838	2641
STIX SGDP + 1KG	3237	843	3531	3284
1KG catalog	193	40	1	1
gnomAD catalog	433	165	88	101

Supplementary Table 3. The frequency of purportedly somatic SVs identified by the PCAWG study considering different SV collections.

	Deletions	Duplications	Inversions
De Novo SV catalog	461	227	10
STIX SGDP	35	13	3
STIX 1KG	6	0	0
STIX SGDP + 1KG	41	13	3
1KG SV catalog	5	0	0
gnomAD SV catalog	19	11	0

Supplementary Table 5. The frequency of purportedly de novo SVs from a large family study. For the STIX counts, samples had at least three supporting reads.

References

1. Zook, J. M. *et al.* An open resource for accurately benchmarking small variant and reference calls. *Nat. Biotechnol.* **37**, 561–566 (2019).
2. Zook, J. M. *et al.* A robust benchmark for detection of germline large deletions and insertions. *Nat. Biotechnol.* (2020) doi:10.1038/s41587-020-0538-8.
3. Forbes, S. A. *et al.* The Catalogue of Somatic Mutations in Cancer (COSMIC). *Curr. Protoc. Hum. Genet.* **Chapter 10**, Unit 10.11 (2008).
4. Sudmant, P. H. *et al.* An integrated map of structural variation in 2,504 human genomes. *Nature* **526**, 75–81 (2015).
5. Collins, R. L. *et al.* A structural variation reference for medical and population genetics. *Nature* **581**, 444–451 (2020).
6. Belyeu, J. R., Brand, H., Wang, H., Zhao, X. & Pedersen, B. S. De novo structural mutation rates and gamete-of-origin biases revealed through genome sequencing of 2,396 families. *bioRxiv* (2020).
7. Jeffares, D. C. *et al.* Transient structural variations have strong effects on quantitative traits and reproductive isolation in fission yeast. *Nat. Commun.* **8**, 14061 (2017).
8. Stefansson, H. *et al.* Large recurrent microdeletions associated with schizophrenia. *Nature* **455**, 232–236 (2008).
